# Supplementary material for: Strain-Induced Form Transition and Crystallization Behavior of the Transparent Polyamide
Source: Polymers (Basel). 2021 Mar 26;13(7):1028. doi: 10.3390/polym13071028 (PMC8036806; doi:10.3390/polym13071028)
Supplement: Supplementary file 1 [file polymers-13-01028-s001.pdf]

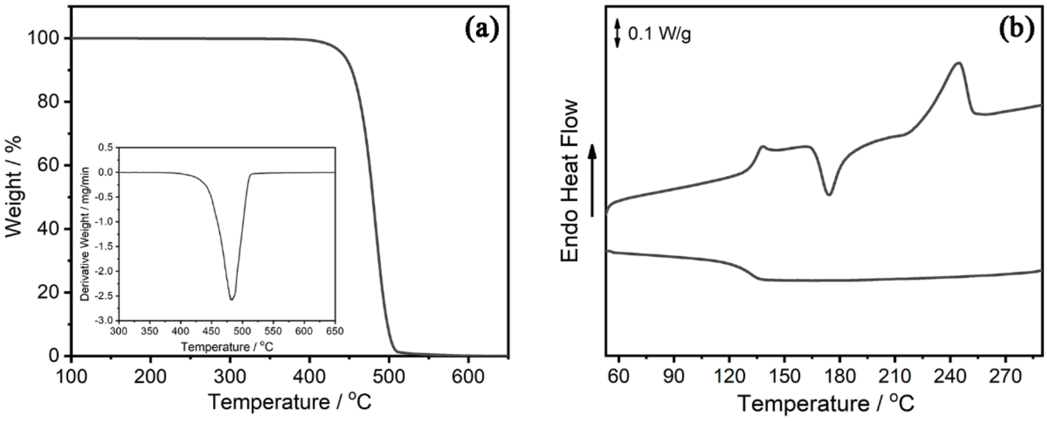

Figure 1. (a) The thermal gravity analysis (TGA) curves of PAPACM12, inset: the derivative thermal analysis (DTA) curves, (b) cooling scans and Secondary heating scans of PAPACM12.

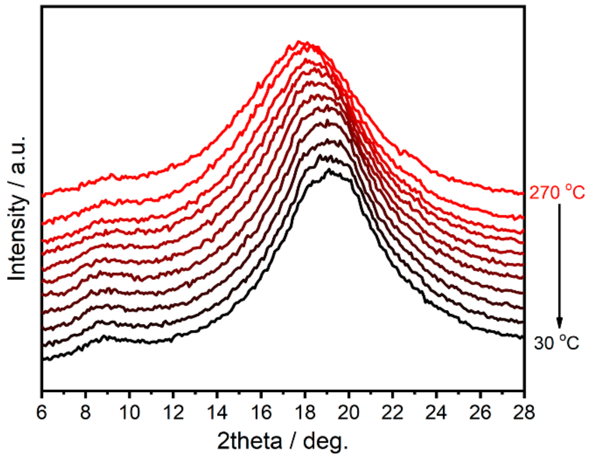

Figure S2. 1D-integrated WAXD profiles in the meridional direction of PAPACM12 upon cooling. Note: WAXD tests were carried out on a Xeuss 2.0 HR SAXS/WAXS system (Xenocs SA, France) with Ni-filtered Cu Kα radiation (λ=0.154 nm).

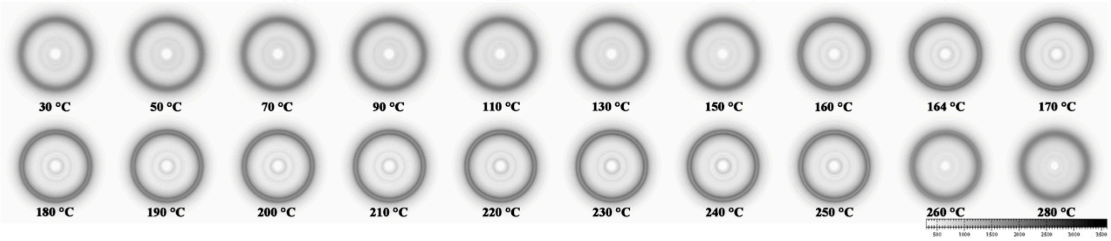

Figure S3. 2D WAXD images of PAPACM12 during heating process with temperatures marked.
